# Approaches to accelerating the study of new antiretrovirals in pregnancy

**DOI:** 10.1002/jia2.25916

**Published:** 2022-07-19

**Authors:** Elaine J. Abrams, Alexandra Calmy, Lee Fairlie, Imelda C. Mahaka, Lameck Chimula, Patricia M. Flynn, John Kinuthia, Landon Myer, Saye H. Khoo, Philippa Musoke, Sheryl Zwerski, Jennifer M. Zech, Shahin Lockman, George K. Siberry

**Affiliations:** ^1^ ICAP at Columbia University Mailman School of Public Health Columbia University New York New York USA; ^2^ Department of Pediatrics Vagelos College of Physicians & Surgeons Columbia University New York New York USA; ^3^ HIV/AIDS Unit Division of Infectious Diseases Geneva University Hospitals Geneva Switzerland; ^4^ Wits RHI University of the Witwatersrand Johannesburg South Africa; ^5^ Pangaea Zimbabwe AIDS Trust Harare Zimbabwe; ^6^ University of North Carolina Project Malawi Lilongwe Malawi; ^7^ Division of Global Women's Health Department of Obstetrics and Gynecology University of North Carolina Chapel Hill North Carolina USA; ^8^ St. Jude Children's Research Hospital Memphis Tennessee USA; ^9^ Department of Research & Programs Kenyatta National Hospital Nairobi Kenya; ^10^ Department of Global Health University of Washington Seattle Washington USA; ^11^ Division of Epidemiology & Biostatistics School of Public Health & Family Medicine University of Cape Town Cape Town South Africa; ^12^ Department of Pharmacology University of Liverpool Liverpool UK; ^13^ Department of Paediatrics and Child Health Makerere University Kampala Uganda; ^14^ Makerere University‐Johns Hopkins University Research Collaboration Kampala Uganda; ^15^ Division of AIDS National Institute of Allergy and Infectious Diseases Rockville Maryland USA; ^16^ Brigham and Women's Hospital Harvard TH Chan School of Public Health Boston Massachusetts USA; ^17^ Office of HIV/AIDS Bureau of Global Health United States Agency for International Development (USAID) Washington DC USA

**Keywords:** antiretrovirals, ARV, clinical trials, pregnancy outcomes, pregnancy, registrational trials

## Abstract

**Introduction:**

Women who are pregnant or who could become pregnant experience delayed access to or underinformed use of important new antiretroviral (ARV) drugs because of traditional drug development processes that ostensibly aim to reduce potential harm but effectively fail to ensure that timely information about safe and effective use in pregnancy is available.

**Discussion:**

The World Health Organization and International Maternal, Pediatric, Adolescent Antiretroviral Clinical Trials Network convened a year‐long workshop on “Approaches to Enhance and Accelerate Study of New Drugs for HIV and Associated Infections in Pregnant Women.” Workshop participants were tasked with defining key principles and optimal approaches to including pregnant women in pre‐ and post‐licensure trials in order to accelerate the availability of pharmacokinetic and safety data for new ARV agents in pregnancy. ARV efficacy in pregnancy and ARV efficacy for prevention of vertical transmission can be extrapolated from proof of efficacy in non‐pregnant adults, provided that drug levels in pregnancy are similar. However, short‐term safety and pharmacokinetics must be studied directly in pregnant women and should be conducted and included in initial licensure for all new ARVs. Accelerating the timeline for completion of pre‐clinical studies is essential for pregnancy short‐term safety and pharmacokinetic studies to be safely completed by the time a drug is licensed. Composite key pregnancy, birth and neonatal outcomes are critical for drugs expected to have broad use, and studies should be initiated at or soon after drug licensure. Teratogenicity risk cannot be feasibly assessed before drug licensure and will depend on robust post‐marketing surveillance systems. With some modifications, these principles will apply to ARVs used for prevention, two‐drug regimens, long‐acting ARVs and ARVs administered through novel delivery systems.

**Conclusions:**

Implementation of the proposed principles and framework will enhance and accelerate the study of new ARVs in pregnancy, resulting in more timely, equitable and informed access to new ARVs for pregnant women.

## 1 INTRODUCTION

Pregnant and breastfeeding women are generally excluded from pre‐licensure trials of new agents and most new medications are approved for use in adult populations without dosing and safety data in pregnancy [1]. Multiple barriers to obtaining pregnancy pharmacokinetic (PK) and safety data have been identified, largely rooted in a historical approach focusing exclusively on protecting the foetus from harm. Delayed completion of pre‐clinical reproductive toxicology studies leads to the exclusion of pregnant women (or women who could become pregnant) from pre‐licensure studies and as justification in pre‐licensure trials for requiring contraception for women of reproductive potential and discontinuing study drug among women participants who become pregnant. The reluctance—fuelled by liability concerns—of sponsors, institutional review boards, investigators, research and regulatory agencies to conduct studies in pregnant women further contributes to the exclusion of this important population and results in delays in obtaining critical PK and safety data [2, 3, 4].

The International Maternal, Pediatric, Adolescent Antiretroviral Clinical Trials Network (IMPAACT) and World Health Organization (WHO) convened a workshop [5] which included discussions on optimal approaches to include pregnant women in pre‐ and post‐licensure trials in order to accelerate the availability of PK and safety data during pregnancy. Here, we build on those discussions and put forward key principles for studying new antiretrovirals (ARVs), as well as other agents, including anti‐infectives, in pregnant women.

## 2 DISCUSSION

### 2.1 Key principles for studying new ARVs in pregnancy

An important first step in optimizing the process for evaluating new ARVs for use in pregnancy is to define what direct evidence is — and is not — required to endorse using new ARVs in pregnancy (Table 1). ARVs that have established therapeutic efficacy (i.e. virologic suppression) in non‐pregnant adults can be considered, by extrapolation, as having therapeutic efficacy in pregnancy, provided that drug exposure in pregnancy is equivalent to therapeutic levels in non‐pregnant adults (Table 1). Similarly, the proven efficacy in achieving virologic suppression in non‐pregnant adults is sufficient for inferring efficacy for prevention of vertical transmission if adequate drug exposures are achieved in pregnancy. Thus, direct clinical evidence of ARV efficacy in pregnancy and of ARV efficacy for prevention of vertical transmission is not needed in order to endorse ARVs for use in pregnancy.

**Table 1:** Key principles and proposed outcomes for the study of new antiretrovirals (ARVs) in pregnancy

*If the agent is efficacious in non‐pregnant adults (viral load suppression) and adequate drug exposures are achieved in pregnancy, then efficacy can be assumed in pregnancy without additional trials*.			
*If the agent is efficacious in non‐pregnant adults (viral load suppression) and adequate drug exposures are achieved in pregnancy, then efficacy for prevention of vertical transmission can be inferred*.			
*All new agents must to be studied in pregnant people for pharmacokinetics/optimal dosing and short‐term safety*.			
*Dedicated pregnancy safety studies assessing pregnancy, birth and infant outcomes should be conducted for all new ARVs with expected broad use in pregnant women and women who may become pregnant*.			
*There is no expectation to have meaningful clinical information about teratogenicity risk before registration; large numbers of observations with exposure at conception/early pregnancy are needed to identify the increased risk of rare events and will only come through post‐marketing surveillance/registries/phase 4 studies*.			
*Once pharmacokinetic/dosing and short‐term safety in pregnancy are determined to be adequate, there should be no restrictions to access during pregnancy once the ARV is licensed*.			

Clinical outcome	Criteria for robust evaluation in pregnancy	Timing of results availability	Comment
Treatment efficacy (viral load suppression)	Not required for any ARV	N/A	Efficacy extrapolated from adult trials
Vertical prevention efficacy	Not required for any ARV	N/A	Inferred based on virologic efficacy
Pharmacokinetic/dosing	Require for ALL new ARVs	Included with application for licensing	Exception: if clear non‐clinical or early clinical signal of reprotoxicity precludes use in pregnancy
Short‐term safety	Require for ALL new ARVs	Included with application for licensing	Exception: if clear non‐clinical or early clinical signal of reprotoxicity precludes use in pregnancy
Composite of: foetal loss (SAB/stillbirth), preterm birth, foetal growth restriction (SGA) and neonatal mortality	Dedicated pregnancy study should be prioritized for ALL new ARVs expected to have high use in women who are or could become pregnant	Study underway at the time of licensing but completed after licensing	Lack of these outcome evaluations should NOT be used to limit the access to ARVs based on pregnancy or pregnancy potential. WHO is leading real‐time processes to regularly assess the priority products for which accelerated development is needed.^a^ Comparative studies with composite outcome likely necessary as described in Brummel et al.^b^
Teratogenicity	Direct, robust clinical evaluation not routine/feasible	Post‐licensure (surveillance and pharmacovigilance)	Preclinical or early clinical signal of reprotoxicity would generally preclude clinical study or use in pregnancy.
Medical disorders of pregnancy (hypertension and gestational diabetes mellitus)	Robust evaluation only if specific concern/signal	Post‐licensure	Collect outcomes in all studies in pregnancy recognizing limited power (small numbers) for robust evaluation. Specific studies may be warranted.
Postnatal growth, development, organ toxicity, neurodevelopment and non‐structural adverse foetal effects	Robust evaluation only if specific concern/signal	Post‐licensure	Comparative studies likely necessary as described in Brummel et al.^b^

Abbreviations: SAB, spontaneous abortion; SGA, small for gestational age.

^a^ World Health Organization. Antiretroviral drug optimization. CADO Reports. WHO; 2022. Available from: https://www.who.int/groups/antiretroviral‐drug‐optimization.

^b^ Brummel et al. [9].

Evidence of appropriate drug exposure and short‐term safety, however, must be directly assessed in pregnancy for all ARVs that could be taken in pregnancy or in women who could become pregnant. Alterations in renal clearance and volume of distribution among other changes in normal pregnancy can result in lower drug exposures with the standard adult dose compared to exposures in non‐pregnant adults [6]. Therefore, direct study of PK and short‐term safety is required in pregnancy (in a relatively small number of participants [<50]) and is informed by dosing and safety profiles from studies in non‐pregnant adults and PK modelling studies when available [1, 7]. The short‐term safety assessments are focused on tolerability and general adverse event profile. Once PK/dosing and short‐term safety in pregnancy are determined to be adequate for an ARV, there should be no restrictions to access to the drug, once licensed, during pregnancy. Pregnancy PK/dosing and safety data are needed for all new ARVs but agents and regimens that will be considered important for treating adults with HIV infection (priority agents identified through the WHO Conference on Antiretroviral Drug Optimization [CADO] [8] process) should be prioritized for gathering these data early in the development process.

There are additional maternal, pregnancy and infant safety outcomes that are extremely important to characterize when considering the use of a new ARV in pregnancy. However, a requirement for robust evaluation of all outcomes before endorsing the use of an ARV in pregnancy is not feasible and may paradoxically limit and delay access to important new ARVs for pregnant women and women who may become pregnant. Thus, PK and short‐term safety should be established as early as possible and before routine use in pregnancy for all new ARVs.

At the same time, it is critically important to conduct dedicated studies in pregnancy to evaluate the composite outcome of preterm delivery, foetal growth restriction and foetal loss along with neonatal mortality for all new ARVs expected to be broadly used in adolescents and adults (priority agents identified through the CADO process [8]). The rationale for conducting these studies as well as innovative approaches to assessing the relationship between new ARVs and pregnancy and birth outcomes are discussed elsewhere in this supplement [9]. Key maternal, pregnancy and infant safety outcomes should also be systematically collected from all pregnant participants (in general ARV prevention and treatment studies or in dedicated pregnancy studies); though the data would typically be too limited to draw definitive conclusions of safety, the results could detect trends or signals that would lead to further evaluation. Notably, some outcomes can only be evaluated with broad uptake after licensure (post‐marketing surveillance). The teratogenic potential of a new ARV — one of the most cited safety concerns by health providers and patients [10] — cannot be meaningfully evaluated before a drug is licensed and in use. Occasionally, teratogenicity will be detected in non‐clinical studies or early clinical studies if the risk is very high. But typically, a specific foetal malformation risk is low and can be detected only after a large number of observations with ARV exposure at conception/early pregnancy — which can only practically come through post‐marketing surveillance [11]. The recognition that only limited assessment of drug teratogenicity is possible before broad use of the agent should not be used as a reason to limit new drug access for pregnant women or women who could become pregnant once dosing and preliminary safety has been determined.

### 2.2 Accelerating the study of new ARV agents in pregnant women: ARVs for treatment

**Figure 1:**
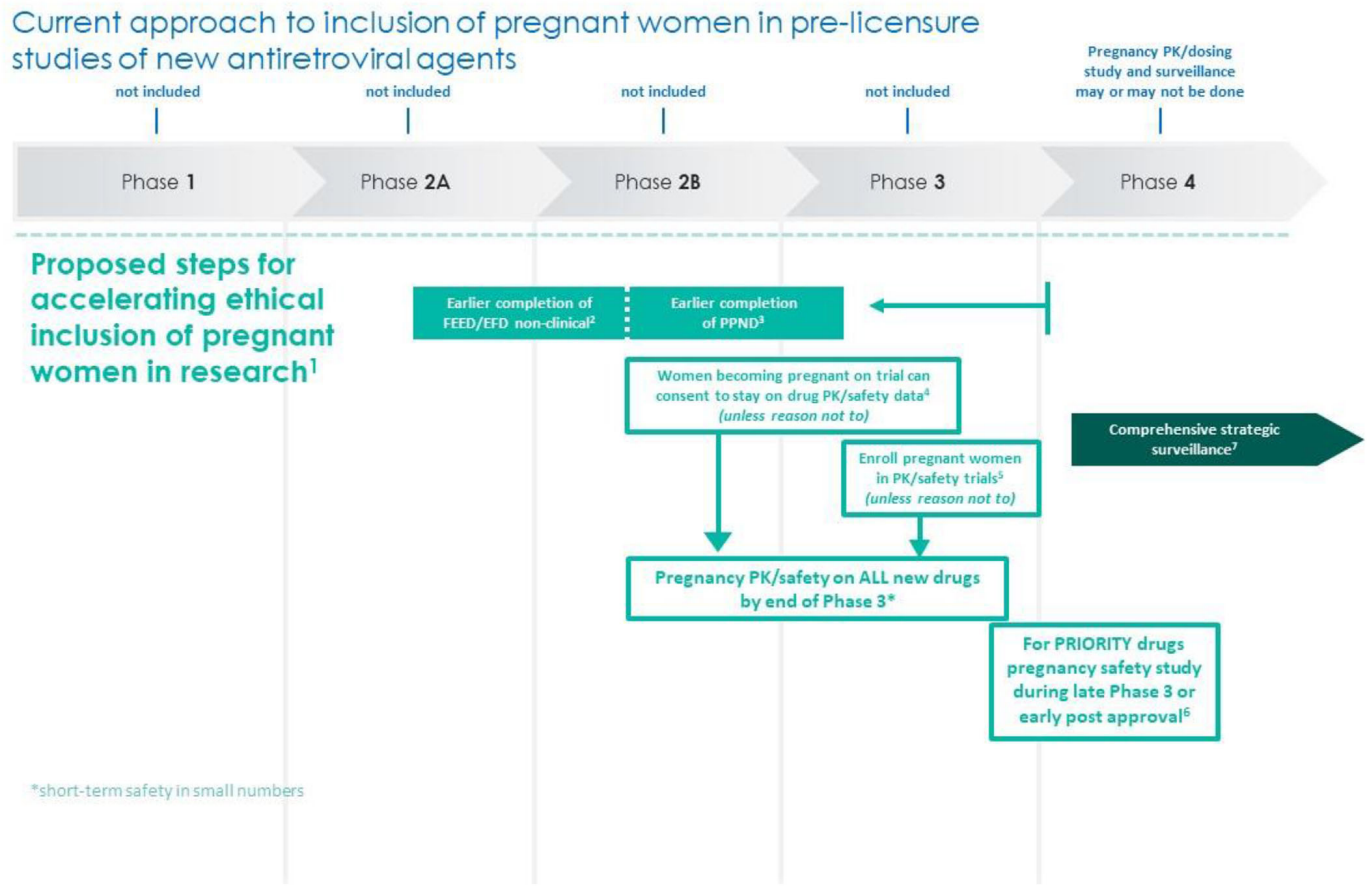
Framework and approaches to expedite the timeline for the study of new antiretrovirals drugs in pregnancy. (1) Involve women of childbearing potential living with HIV from the identification of research questions through the study design, recruitment, conduct and dissemination of results. (2) Perform non‐clinical developmental and reproductive toxicology studies (DART) earlier during drug development for all new HIV agents. Fertility and early embryonic development (FEED) and embryo‐foetal development (EFD) studies should be completed during or no later than the end of the phase 2 registrational trials. (3) Pre‐ and postnatal development (PPND) studies should be completed during early phase 3 or no later than the end of phase 3 registrational trial. (4) Women who become pregnant in registrational trials should be given the option to make an informed choice to stay on study drug once early non‐clinical FEED and EFD studies are completed, with no negative signals and dosing is established in non‐pregnant people. (5) Enrol pregnant women in specific studies to determine pharmacokinetic (PK) and preliminary safety as soon as late non‐clinical PPND studies are completed with no negative signals for all new HIV agents. (6) Investigate adverse maternal, pregnancy and birth outcomes through dedicated pregnancy safety studies for all new priority HIV agents identified through CADO as soon as dosing is confirmed. (7) Expand systematic and rigorous active safety surveillance studies to enable systematic and rapid detection of adverse birth outcomes and rare events, such as birth defects associated with exposure to antiretrovirals during pregnancy.

Approaches to expedite the timeline for the study of new ARVs in pregnancy are delineated in Figure 1. Currently, at the time of licensure, new ARVs have preclinical study data available (regulatory requirement) but rarely have information in the label about clinical studies in pregnancy. In almost all cases, PK and short‐term safety studies in pregnancy are completed well *after* licensure. Preclinical developmental and reproductive toxicology should be completed earlier in the drug development cycle [2]. More specifically, non‐clinical studies (fertility and early embryonic development and embryo‐foetal development) should be completed during or no later than the end of the phase 2 registrational trial; pre‐ and postnatal development studies should be completed at or near the beginning of the phase 3 registrational trials. Approaches to facilitate more rapid completion of these studies are discussed in other papers in this supplement [2, 12], but this accelerated timeline would enable short‐term safety and PK pregnancy data to be available by the end of the phase 3 registrational trial and thus included in the regulatory submission for licensure. It will not be feasible to complete a dedicated study of composite key foetal/pregnancy/neonatal outcomes by the time of licensure but having that evaluation underway at the time of licensure would expedite the availability of this critical information.

### 2.3 Accelerating the study of new ARV agents in pregnant women: ARVs for prevention

Most new agents are now being developed and studied for both prevention and treatment indications. The risk of HIV acquisition appears to be somewhat higher in pregnant and breastfeeding women compared to non‐pregnant women; furthermore, pregnant women have signalled interest in participating in HIV prevention research, highlighting that preventing HIV infection will protect themselves and their infants [13, 14, 15]. Similar to the principles articulated for ARV treatment studies, if efficacy of a new agent for prevention is demonstrated in non‐pregnant individuals and drug exposure in pregnancy is similar, prevention efficacy from non‐pregnancy trials can be extrapolated to pregnant populations. At the same time, while many of the principles and approaches defined for ARV treatment studies apply equally to prevention studies, the risk–benefit ratio for women who become pregnant or enrol in pre‐licensure prevention trials while pregnant is likely to be somewhat different from women living with HIV considering treatment options. Considerations need to include existing safety data on the new agent, alternative prevention agents and approaches, motivation for study participation, individual risk perception of HIV acquisition, as well as perception of risk to the mother, her baby and the pregnancy [16, 17]. Currently, one large prevention study of a new ARV, lenacapravir, is enrolling sexually active women of reproductive potential [18]. This study offers unique opportunities to (1) rapidly determine PK and safety of these new agents if women who become pregnant on study are given the option to stay on study drug and (2) once PK is determined, given sufficient number of incident pregnancies likely to occur, permit randomized comparison of pregnancy‐specific safety outcomes of the new agent. Given the potentially large number of pregnancies among participants in this trial, the timeline for obtaining critical data to inform safe use of this agent in pregnancy could be accelerated. Whether data collected in HIV‐negative women can be extrapolated to women living with HIV is an open question. While drug metabolism is likely to be the same in women with and without HIV infection, consideration will need to be given to the ARV(s) used in a treatment regimen, potential drug–drug interactions and a potential need for small confirmatory PK studies in pregnant women living with HIV.

### 2.4 Special considerations: long‐acting formulations, dual regimens and biologicals

#### 2.4.1 Long‐acting formulations

The pipeline of anti‐HIV drugs covers a wide range of long‐acting (LA) formulations (injectables, implants, oral and ring), which have been developed for both prevention and treatment. The expected benefits of using LA strategies are immense: improved adherence and maintenance of effective drug levels over long periods of time could translate into decreased risk of HIV acquisition, improved survival for individuals living with HIV and decreased risk of sexual and vertical transmission. To ensure early universal access to these innovations, it is critical that pregnant women or women of childbearing age are included in clinical trials of these new agents. To date, there are few data on LA agents in pregnancy. However, it is expected that once LA agents become available, women will likely become pregnant on these formulations. The benefits using or continuing to use these LA agents will have to be balanced against the paucity of PK and safety data in pregnancy.

Scenarios of how and when to study new LA agents, however, are not substantially different from the principles articulated for traditional ARVs (Figure 1). PK and safety should be determined as soon as early non‐clinical trials are completed (assuming no negative signals), and once non‐pregnant adult dosing is determined, women who become pregnant during phase 3 trials should be given the choice to remain on drugs. As noted above, the risk/benefit analysis of continuing study drug will likely vary by indication (prevention vs. treatment) but, in contrast to traditional ARVs, drug levels (and likely foetal exposure) of LA agents can remain high for many months after discontinuing use. Finally, dedicated pregnancy safety studies should be done for all priority agents with anticipated broad use, whether for a preventive and/or therapeutic indication.

#### 2.4.2 Dual‐ARV regimens

These are increasingly prescribed for HIV treatment, both for treatment‐naïve and treatment‐experienced non‐pregnant adults, and the group considered use in pregnancy. In general, provided that both drugs are known to be safe during pregnancy, pregnancy PK/dosing data are available and adequate for both agents and the efficacy of the dual combination has been demonstrated in non‐pregnant adults, there is no need to study the efficacy in pregnancy. For example, there are adequate data on DTG/3TC dual therapy to suggest that it is safe to use in pregnancy and that there would be no reason to add a third ARV if a virally suppressed person on the dual regimen became pregnant. Adding a third drug may compromise safety and tolerance without improving efficacy.

#### 2.4.3 Monoclonal antibodies

Monoclonal antibodies (mAbs) represent a distinct category of drugs when compared to direct‐acting (small molecule) ARVs. Only one drug of this category is FDA approved for the treatment of difficult‐to‐treat HIV infection: ibalizumab. US FDA category for pregnancy use was not assigned, nor is there any recommendation from the manufacturer. However, based on the findings of significant immunosuppression (CD4^+^ T cell and B cell lymphocytopenia) among infant cynomolgus monkeys born to mothers who received ibalizumab during pregnancy, a pregnancy warning was issued noting that the use of the agent during pregnancy may cause reversible immunosuppression in infants exposed to the drug in utero [19].

It is possible to anticipate placental transfer for all mAbs. Placental transfer of human IgG depends on the IgG subclass, maternal serum concentrations, birthweight of the newborn and gestational age, and generally increases as pregnancy progresses. Transfer of IgG from mother to foetus begins as early as 13 weeks’ gestation, and transport occurs in a linear fashion as pregnancy progresses, with the greatest amount transferred in the third trimester.

In comparison to ARVs, there are fewer required non‐clinical studies to support clinical trials of mAbs in pregnant women [2]. For example, a reduced toxicology program was conducted for REGN‐COV2, including a 4‐week repeat‐dose toxicity study in cynomolgus monkeys and cross‐reactivity studies with healthy human and monkey tissues and human foetal tissues with no deleterious signal reported. In addition, the target of REGN‐COV2 is specific for viral proteins and, therefore, unlikely to affect foetal development. In summary, biologicals, including large human IgG developed to prevent or treat HIV infection, may not need similar strategies to assess their safe use during pregnancy. Furthermore, there is increasing experience with mAbs in pregnancy for other conditions, which may inform the understanding of potential side effects associated with the use of these molecules in pregnancy. Currently, mAbs are not a high priority for treatment in pregnant women with HIV but are of great interest for potential use in infants for prevention of breastfeeding transmission [20]. In addition to short‐ and long‐term safety, efficacy for breastfeeding transmission prevention would need to be directly evaluated in a clinical trial.

## 3 CONCLUSIONS

In this paper, we describe the principles and framework to enhance and accelerate the study of new ARVs in pregnancy. The proposed approaches were developed with the aim of addressing well‐described barriers to inclusion of pregnant women in pre‐licensure studies and to provide an actionable, practical roadmap to obtaining PK and preliminary safety data by the time of drug registration, and pregnancy‐specific safety data for prioritized agents soon thereafter. While specifically developed with the aim to accelerate the study of ARV agents for prevention and treatment of HIV infection, these approaches can be easily applied across a broad range of agents commonly use to treat or prevent other conditions in pregnancy, including hepatitis, tuberculosis, malaria, COVID‐19 and non‐infectious conditions.

It is noted, however, that there are regulatory limitations, funding challenges, resistance of local ethical review boards and investigators’ hesitancy—among other barriers—that will need to be addressed to ensure that pregnant women are included in pre‐licensure trials and that the necessary pregnancy‐specific safety trials of high‐priority agents are conducted. Early engagement with pharmaceutical companies will be critical to encourage early completion of non‐clinical studies and to make pregnancy studies a critical component of drug development plans for key agents [21]. Additional guidance from regulators to enable and prioritize the study of new drugs in pregnant women will be pivotal to any efforts to accelerate the traditionally delayed timeline. CADO will continue to convene a formal process to anticipate priority products that should receive accelerated support, and the WHO has committed to continue to host the technical dialogue to ensure development and updating of appropriate tools and policies to support the implementation of accelerated approaches to this research. Furthermore, all key stakeholders, including researchers, study sponsors, ethics committees, publishers and particularly community members, must take every opportunity to ask why pregnant women were excluded from the study until we see a substantial shift in the landscape from the current exclusionary approaches to the more inclusive, accelerated principles and framework that we and others have put forward [3].

## COMPETING INTERESTS

AC has received research funding from MSD. PMF serves as a safety committee member for Merck. SK has received research funding support from ViiV, Gilead and Merck, and speakers honoraria and consultancy from Merck, Thera Technologies and ViiV. All other authors declare no competing interests.

## AUTHORS’ CONTRIBUTIONS

EJA, AC and GKS developed the concept and outline for this commentary. First draft of the manuscript was written by EJA, GKS, AC and LF. All authors (EJA, AC, LF, ICM, LC, PMF, JK, LM, SK, PM, SZ, JMZ, SL and GKS) participated in the workgroup and provided comments and edits on the initial draft and approved the final manuscript.

## DISCLAIMER

The views expressed in this article are those of the authors and do not necessarily reflect the views of the U.S. Agency for International Development, U.S. National Institutes of Health, U.S. Food and Drug Administration or the U.S. Government.
